# Machine Learning-Driven Prediction and Optimization of Thermoelectric Transport Properties in Ce-Doped CaMnO_3_ Ceramics

**DOI:** 10.1021/acsomega.6c09057

**Published:** 2026-10-20

**Authors:** M. Gürsul, İ Uluocak, B. Özçelik, Sh Rasekh, M. A. Madre, A. Sotelo

**Affiliations:** † Department of Physics, Faculty of Science and Letters, 37506 Çukurova University, 01330 Adana, Turkey; ‡ Department of Mechanical Engineering, Ceyhan Engineering Faculty, Çukurova University, 01330 Adana, Turkey; § 16765 INMA (CSIC-Universidad de Zaragoza), Maria de Luna, 50018 Zaragoza, Spain

## Abstract

Oxide-based thermoelectric materials are promising candidates for high-temperature waste heat recovery owing to their excellent thermal and chemical stability. Nevertheless, optimizing their thermoelectric performance remains challenging because electrical resistivity and the Seebeck coefficient exhibit complex, nonlinear, and composition-dependent relationships. In this work, experimental measurements combined with data-driven machine learning approaches were employed to predict and optimize the high-temperature thermoelectric behavior of Ce-doped CaMnO_3_ (Ca_1–*x*_Ce_*x*_MnO_3_, *x* = 0.02–0.10) ceramics using Support Vector Machine (SVM), Multilayer Perceptron (MLP), eXtreme Gradient Boosting (XGBoost), and a hybrid Convolutional Neural Network–Long Short-term Memory (CNN–LSTM) architecture. The models were trained and validated using experimentally measured electrical resistivity (ρ) and Seebeck coefficient (*S*) data collected over the temperature range of 50–800 °C. Among the investigated approaches, the hybrid CNN–LSTM model, which uniquely combines spatial feature extraction with sequential learning to resolve complex nonlinear transport behaviors that conventional machine-learning models fail to capture accurately, exhibited the highest predictive performance, achieving coefficients of determination (*R* ^2^) of 0.9982 for electrical resistivity and 0.9987 for the Seebeck coefficient. Permutation feature importance analysis further revealed that the Ce concentration has a stronger influence on the transport properties than the operating temperature. To identify the optimum operating conditions, 150,000 Monte Carlo simulations were performed using the trained CNN–LSTM model to generate continuous multidimensional response surfaces. The optimization results identified a favorable thermoelectric region at a Ce content of *x* = 0.05–0.06 and temperatures between 350 and 500 °C, where the simulated power factor exceeded 0.35 mW/K^2^m. These results demonstrate the potential of hybrid deep learning for data-driven analysis and optimization of oxide-based thermoelectric materials under the investigated experimental conditions.

## Graphical abstract

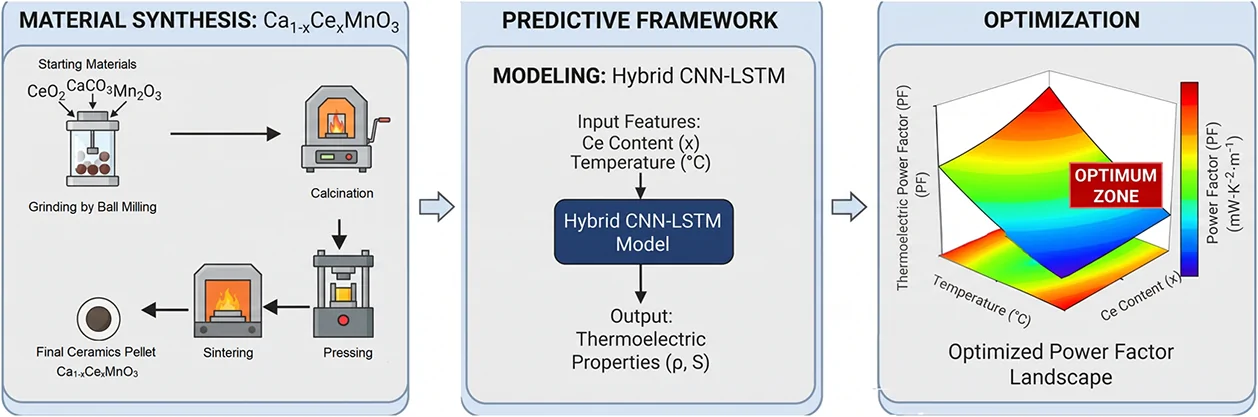

## 1 Introduction

The continuous consumption of fossil fuels and escalating energy demands have made the development of sustainable energy technologies a critical priority. A significant fraction of primary energy is dissipated as industrial waste heat, driving the urgent need for efficient thermal-to-electrical recovery solutions.^1,2^ Among various strategies, thermoelectric (TE) energy conversion has attracted considerable attention due to its ability to directly convert thermal gradients into electricity without moving parts or harmful fluids, positioning it as a promising component of future carbon-neutral energy systems.^3^ The efficiency of a thermoelectric material is commonly evaluated using the dimensionless figure of merit, ZT, defined as,

1 $$ZT=\frac{S^{2}T}{\rho k}$$

where *S* is the Seebeck coefficient, ρ is the electrical resistivity, *k* is the total thermal conductivity, and *T* is the absolute working temperature. According to this relationship, high thermoelectric performance requires a large power factor, PF, (*S* ^2^/ρ) together with low thermal conductivity. Because these transport parameters are strongly coupled, various optimization strategies, including carrier concentration engineering,^4^ defect manipulation,^5^ nanostructuring,^6^ band structure modification,^7^ and elemental substitution,^8^ are widely employed. While traditional chalcogenides and intermetallics (Bi_2_Te_3_, PbTe, SiGe) exhibit high ZT near ambient temperatures,^9^ their high-temperature deployment is severely restricted by thermal instability and toxicity.^10^ Consequently, oxide-based thermoelectric materials have gained significant interest due to their superior chemical stability and environmental compatibility.^11^ Among them, perovskite-structured oxides offer flexible frameworks for systematic property tuning.^12^ Although developing efficient n-type oxides remains challenging relative to p-type counterparts like Ca_3_Co_4_O_9_,^13^ orthorhombic CaMnO_3_ has emerged as a premier high-temperature n-type candidate owing to its exceptional oxidation resistance and structural durability.^11,14^ However, pristine CaMnO_3_ suffers from high electrical resistivity,^15^ necessitating aliovalent substitution at the Ca- or Mn-sites to tailor carrier concentration, defect chemistry, and lattice distortion for optimized performance.^16–18^ The substitution of higher-valent Ce^4+^ for Ca^2+^ leads to the formation of Mn^3+^ states, effectively tailoring the Mn^3+^/Mn^4+^ ratio and governing carrier concentration and electron hopping. Furthermore, the ionic radius mismatch introduces local lattice distortions that enhance phonon scattering while preserving the structural stability of the perovskite framework.^19,20^ Given that the thermoelectric properties of Ce-doped CaMnO_3_ are highly sensitive to dopant concentration and operating temperature, conventional trial-and-error optimization is time-consuming and costly. Consequently, data-driven computational techniques capable of accurately predicting material properties have gained significant attention. Machine learning algorithms can learn nonlinear correlations between material composition, processing conditions, and functional properties, and have been successfully applied to a wide range of materials science applications, including crystal structure prediction, phase identification, battery materials, catalysts, superconductors, and thermoelectric materials.^21–27^

In thermoelectric research, machine learning (ML) techniques have attracted increasing attention for predicting thermoelectric properties and improving material performance.^28,29^ ML models can learn complex relationships between material characteristics and thermoelectric behavior, reducing the need for extensive experimental trials. Recent studies have demonstrated that thermoelectric performance can be predicted using readily available structural and electrical parameters.^30^ In addition, ML has been applied to identify oxide materials with low thermal conductivity, demonstrating its potential for the screening and design of new thermoelectric oxides.^31^ These studies highlight the growing role of data-driven methods in thermoelectric material prediction, optimization, and discovery. Compared to conventional statistical models, modern machine learning algorithms can effectively model highly nonlinear relationships without requiring explicit physical equations. Among the most widely used regression algorithms, support vector machine (SVM), multilayer perceptron (MLP), and eXtreme Gradient Boosting (XGBoost) have demonstrated excellent predictive performance in numerous materials science applications.^32–34^ SVM is well-suited for nonlinear regression through kernel-based learning, MLP effectively models complex multidimensional relationships using artificial neural networks, whereas XGBoost efficiently captures nonlinear feature interactions through ensemble learning techniques. More recently, deep learning has emerged as one of the most powerful approaches for materials informatics owing to its capability to automatically learn hierarchical feature representations from large data sets. In particular, hybrid Convolutional Neural Network–Long Short-Term Memory (CNN–LSTM) architectures combine the advantages of convolutional layers for extracting local feature patterns with the memory capability of LSTM networks for modeling complex feature dependencies.^35^ This hybrid framework has demonstrated superior predictive performance in many nonlinear regression problems and, therefore, offers considerable potential for modeling the strongly coupled transport behavior of thermoelectric materials. Despite the growing application of machine learning in materials science, studies employing hybrid deep-learning models for oxide thermoelectric materials remain relatively limited. In particular, comprehensive predictive investigations on Ce-doped CaMnO_3_ ceramics are still scarce, although their thermoelectric properties exhibit highly nonlinear dependencies on the dopant concentration and operating temperature. Developing accurate predictive models for such systems would not only reduce the need for extensive experimental investigations but also facilitate the identification of the optimum operating conditions and material compositions. Furthermore, Ce-doped CaMnO_3_ was specifically chosen as a benchmark system in this work because n-type perovskite oxides provide exceptional thermal and chemical stability for high-temperature waste heat recovery, thereby overcoming the toxicity and degradation issues of traditional intermetallics (such as Bi_2_Te_3_). Additionally, the complex and highly nonlinear interplay between dopant concentration and transport properties, which is governed by Mn^3+^/Mn^4+^ mixed-valence states and the double-exchange mechanism, makes this material an ideal and rigorous physical platform for benchmarking the predictive capabilities of advanced machine learning and stochastic optimization algorithms.

In this study, the thermoelectric properties of Ce-doped CaMnO_3_ ceramics were modeled using four different machine learning approaches, namely, support vector machine (SVM), multilayer perceptron (MLP), extreme Gradient Boosting (XGBoost), and a hybrid CNN–LSTM deep learning architecture. These specific algorithms, and particularly the hybrid CNN–LSTM framework, were selected because they uniquely combine local pattern extraction with sequential memory, offering superior advantages over traditional experimental trial and error and simple statistical models in capturing strongly coupled transport phenomena. Utilizing dopant concentration and operating temperature as input features to predict electrical resistivity and Seebeck coefficient as output targets, this work establishes the first dedicated predictive machine-learning model for the Ce-doped CaMnO_3_ system. The experimental data of electrical resistivity and Seebeck coefficient used in this study were previously reported by our group in ref 19. The present work focuses on machine-learning modeling and optimization of these data, which have not been reported previously. While our previous work established these experimental synthesis protocols and fundamental physical characteristics, the present study introduces a comprehensive, data-driven materials informatics framework. Rather than relying solely on conventional experimental trial and error, this work applies advanced machine learning algorithms to model and map the complex and highly nonlinear dependencies among dopant concentration, temperature, and thermoelectric transport properties. The predictive performances of all models were systematically evaluated and compared by using statistical performance metrics. Furthermore, by integrating the trained deep-learning model with continuous response surfaces and large-scale Monte Carlo simulations, this study goes beyond standard experimental observation to systematically explore the unsynthesized compositional landscape and precisely identify optimal performance regions. The proposed framework demonstrates the effectiveness of hybrid deep learning as a reliable and efficient tool for predicting the thermoelectric properties of oxide ceramics and accelerating the optimization of high-performance thermoelectric materials through data-driven material engineering.

## 2 Materials and Methods

### 2.1 Experimental Procedure

Polycrystalline Ca_1–*x*_Ce_*x*_MnO_3_ ceramics with nominal compositions of *x* = 0.00, 0.02, 0.04, 0.06, 0.08, and 0.10 were synthesized by the conventional solid-state reaction method using high-purity commercial CaCO_3_ (≥99.0%, PanReac), Mn_2_O_3_ (≥99.0%, Aldrich), and CeO_2_ (≥99.0%, Aldrich) powders as starting materials. The precursor powders were accurately weighed according to the stoichiometric compositions using a high-precision electronic balance and thoroughly mixed by ball milling in double-distilled water at 300 rpm for 30 min to achieve homogeneous mixing. The resulting slurry was dried using an infrared (IR) drying system, manually ground into fine powders, and calcined in alumina crucibles through a two-step heat treatment at 950 and 1050 °C for 12 h, with intermediate grinding after each calcination step to improve phase homogeneity. The calcined powders were uniaxially pressed into pellets (3 × 3 × 15 mm^3^) under a pressure of 400 MPa and subsequently sintered at 1300 °C for 24 h in air. After sintering, the samples were furnace-cooled to room temperature. The surface morphology and microstructure of the sintered samples were investigated using a Field Emission Scanning Electron Microscope (FESEM, Zeiss Merlin). Prior to SEM analysis, the sample surfaces were carefully ground and polished to obtain a smooth surface. The micrographs were acquired using an accelerating voltage of 15.00 kV and a probe current of 600 pA, with the working distance (WD) adjusted to optimal focus (typically ranging around 5.5 mm) at a magnification of 2.00 kX. The thermoelectric properties were experimentally measured using a Linseis LSR-3 measurement system operating with the conventional dc four-probe technique under a helium (He) atmosphere. Electrical resistivity (ρ) and Seebeck coefficient (*S*) were simultaneously measured over the temperature range of 50–800 °C during the heating cycle, with predefined temperature steps and thermal equilibration intervals implemented at each target point to ensure steady-state stability. The measurements utilized spring-loaded thermocouple probes and standard nickel-based contact configurations for reliable electrical and thermal coupling. The power factor was calculated from S and ρ data. The measurement uncertainties were approximately 4% for electrical resistivity and 4% for the Seebeck coefficient, resulting in an estimated uncertainty of approximately 12% for the calculated power factor.^36^ The experimentally measured thermoelectric data constituted the data set employed in the present machine learning study. Temperature, Ce concentration, electrical resistivity, Seebeck coefficient, and calculated power factor values were used as the input data for model development and performance evaluation. Note that while the experimental characterization covers the entire series including the pristine sample (*x* = 0.00), the machine learning data set and subsequent predictive modeling were strictly restricted to the Ce-doped compositional range of *x* = 0.02–0.10. The undoped *x* = 0.00 composition was intentionally excluded from the training because CaMnO_3_ is a high-resistance semiconductor (2 × 10^5^ mΩ·cm), whereas even a tiny amount of Ce doping (*x* = 0.02) triggers a drastic semiconductor-to-metal transition that drops the resistivity by orders of magnitude via the double-exchange mechanism. The preprocessing of the experimental data and the implementation of the machine learning algorithms are described in the following section.

### 2.2 Methodology: Machine Learning Models

#### 2.2.1 Multilayer Perceptron (MLP)

The Multilayer Perceptron (MLP) is a fundamental feed-forward artificial neural network architecture. It consists of an input layer, one or more hidden layers, and an output layer. Each node in a layer is fully connected to the nodes in the subsequent layer through adjustable weights and biases. The learning process involves the optimization of a loss function *J*(θ) via backpropagation and gradient descent. For each neuron *j* in a hidden layer, the output *y_j_* is defined as

2 $$y_{j}=\phi \left(\Sigma_{i=1}^{n}w_{\mathrm{ij}}x_{i}+b_{j}\right)$$

where *x_i_* represents the input signals, *w_ij_* denotes the weights, *b_j_* is the bias, and ϕ is a nonlinear activation function (e.g., ReLU or Sigmoid) which allows the model to capture nonlinear relationships within the data set.

#### 2.2.2 Support Vector Machine (SVM)

The support vector machine (SVM) is a robust supervised learning algorithm used for both classification and regression tasks. The primary objective of SVM is to identify an optimal hyperplane in a high-dimensional feature space that maximizes the margin between different classes or minimizes the deviation for regression. To handle nonlinear data distributions, a kernel function *K* (*x_i_*, *x_j_*) (such as the radial basis function (RBF)) is employed to map the input features into a higher-dimensional space where linear separation becomes feasible. The optimization problem is formulated as

3 $$\begin{array}{l} \min\\ w,b \end{array}\frac{1}{2}{∥w∥}^{2}+C\Sigma_{i=1}^{n}\xi_{i}$$

subject to the constraints of the specific task, where *C* is the regularization parameter and ξ*_i_* represents slack variables for handling outliers.

#### 2.2.3 Long Short-Term Memory (LSTM)

Long short-term memory (LSTM) networks are a specialized type of recurrent neural network (RNN) designed to overcome the vanishing gradient problem in sequence modeling. LSTMs maintain a persistent cell state (c*_t_*) and utilize a gating mechanism to regulate the flow of information. This mechanism consists of three primary components:

Forget gate (*f*_t_): decides which information from the previous state should be discarded.

Input gate (*i*_t_): determines which new information will be stored in the cell state.

Output gate (*o*_t_): controls the final output based on the filtered cell state.

This architecture enables the model to learn long-range temporal dependencies, making it highly effective for time series forecasting and sequential data analysis.

#### 2.2.4 Hybrid CNN–LSTM Model

The hybrid CNN–LSTM model combines the feature extraction capability of convolutional neural networks (CNNs) with the temporal learning ability of LSTM networks. The CNN layers automatically extract representative local features from the input data, while the LSTM layer models the sequential relationships among the features. By integrating both architectures, the CNN–LSTM model improves the prediction of complex nonlinear behaviors and has demonstrated excellent performance in regression and time-series applications.

### 2.3 Model Evaluation and Data Partitioning

The statistical reliability and generalization capability of the developed multioutput frameworks were enforced using a rigorous data partitioning and cross-validation strategy, as implemented in our MATLAB Code. Given the highly coupled and nonlinear nature of thermoelectric transport properties, standard random train-test splits can introduce structural data leakage or yield overoptimistic performance metrics that fail under true experimental validation. To mitigate these risks, a 5-fold cross-validation scheme was systematically executed across the entire preprocessed data set. The experimental data set, containing two independent input features, Ce content (%) and temperature (°C), and two continuous target outputs, electrical resistivity (ρ) and Seebeck coefficient (*S*), with 775 data points for each, was partitioned in the MATLAB platform using the cvpartition class with the *K* Fold parameter set to 5. This configuration divides the total observations into five distinct, nonoverlapping subsets of equal size. During each iterative loop of the cross-validation process, four subsets are concatenated to establish the training matrix, while the remaining single fold is strictly reserved as an independent testing matrix for validation. To ensure absolute statistical reproducibility across all baseline architectures, the random number generator was anchored with a fixed seed utilizing rng(42)^42^ code prior to partitioning. The data flow within the cross-validation partition is tailored to satisfy the distinct mathematical input structures required by the deep learning and conventional machine-learning models. For the proposed hybrid CNN–LSTM framework, the standardized input matrices are dynamically converted into sequential cell arrays via the num2cell function, allowing the spatial-temporal network to parse each sample slice correctly. Conversely, the benchmark configurations, including Support Vector Regression (SVR), Multilayer Perceptron (MLP), and eXtreme Gradient Boosting (XGBoost), digest the data partitions in their native matrix formats. During each validation fold, the network outputs are inversely transformed using the “stored actual training statistics” to revert the predictions from normalized dimensions back to their physical engineering units (mohm·cm and μV/K). The cross-validation performance of each architecture was quantitatively logged across all folds by storing the denormalized actual and predicted matrices into continuous evaluation arrays. This comprehensive validation loop guarantees that the subsurface response mappings and subsequent power factor calculations are extracted from a model structure that has been thoroughly cross-verified against localized experimental variances. The accuracy of the models was quantified using three widely recognized statistical metrics: the coefficient of determination (*R* ^2^), mean absolute error (MAE), and root-mean-square error (RMSE). These metrics provide a comprehensive assessment of the models’ precision and error distribution:

Coefficient of determination (*R* ^2^): measures the proportion of variance in the dependent variable that is predictable from the independent variables. An *R* ^2^ value closer to 1 indicates a superior fit.

4 $$R^{2}=1-\frac{\sum_{i=1}^{N}{\left(y_{i}-{ŷ}_{i}\right)}^{2}}{\sum_{i=1}^{N}{\left(y_{i}-\mathrm{y̅}\right)}^{2}}$$

Mean absolute error (MAE): represents the average of the absolute differences between the predicted values and the actual observations, providing a linear measure of the average error magnitude.

5 $$\mathrm{MAE}=\frac{1}{N}\Sigma_{i=1}^{N}\left|y_{i}-{ŷ}_{i}\right|$$

Root-mean-square error (RMSE): calculates the square root of the average of squared differences between prediction and actual observation. Due to the squaring of error terms, RMSE is more sensitive to outliers than MAE.

6 $$\mathrm{RMSE}=\sqrt{\frac{1}{N}\Sigma_{i=1}^{N}{\left(y_{i}-{ŷ}_{i}\right)}^{2}}$$

where *y*_*i*_ and *ŷ*_*i*_ denote the measured and predicted values, respectively, *y̅* is the mean of the measured values for the *i*th sample, and *N* is the total number of samples.

## 3 Results and Discussion

### 3.1 Experimental Characterization

The FESEM micrographs of the Ca_1–*x*_Ce_*x*_MnO_3_ ceramics with *x* = 0.00, 0.04, and 0.10 recorded at 2000× are presented in Figure 1. A comprehensive experimental investigation of the entire composition series (*x* = 0.00, 0.02, 0.04, 0.06, 0.08, and 0.10), including structural, microstructural, electrical, magnetic, and thermoelectric characterizations, has been reported in our previous work.^19^ As confirmed by X-ray diffraction (XRD) analyses in ref 19, all synthesized samples successfully formed a single-phase CaMnO_3_ structure, verifying the successful incorporation of Ce into the lattice. Since the primary objective of the present work is to predict thermoelectric properties using machine learning, only representative FESEM micrographs are included in Figure 1 for the sake of completeness and consistency in the experimental characterization. The selected images correspond to the undoped, intermediate, and highest Ce-doped compositions (*x* = 0.00, 0.04, and 0.10), providing a clear overview of the microstructural characteristics of the synthesized ceramics. As observed from the micrographs, all samples exhibit the characteristic morphology typically reported for polycrystalline CaMnO_3_ ceramics.^37,38^ The microstructure consists of well-developed polygonal grains with clearly distinguishable grain boundaries, indicating successful grain formation during the conventional solid-state sintering process. The grains are uniformly distributed throughout the microstructure, and no abnormal grain growth, microcracks, or noticeable morphological heterogeneity was observed. A small amount of residual porosity is present across all compositions, which is typical for conventionally sintered CaMnO_3_-based ceramics. The incorporation of Ce slightly modifies the grain morphology without significantly altering the overall microstructural characteristics of the CaMnO_3_ systems. Compared to the undoped sample, the ceramic containing *x* = 0.04 exhibits a modest increase in average grain size, suggesting that a moderate Ce addition slightly promotes grain growth during the sintering process. Furthermore, the grain boundaries appear to be well-connected, indicating good intergranular contact. However, further increasing the Ce concentration to *x* = 0.10 leads to a slight reduction in the average grain size, resulting in a relatively finer microstructure. This behavior suggests that while moderate Ce substitution favors grain growth, excessive Ce addition tends to suppress further grain coarsening. Despite these subtle variations in grain size, the overall morphology remains essentially unchanged with an increasing Ce content. All compositions retain dense polycrystalline microstructures with similar grain shapes and homogeneous grain distribution. Therefore, the FESEM observations indicate that Ce substitution has only a limited influence on the microstructural evolution while preserving its typical ceramic morphology of the host lattice. The observed differences in grain size are relatively small, implying that any significant changes in the electrical and thermoelectric properties are more likely to originate from the electronic effects of Ce substitution rather than from drastic microstructural modifications. Consequently, this structural homogeneity ensures that the variations in electronic transport properties can be confidently modeled by data-driven algorithms primarily as functions of temperature and dopant concentration rather than being convoluted by erratic microstructural anomalies.

**Figure 1:**
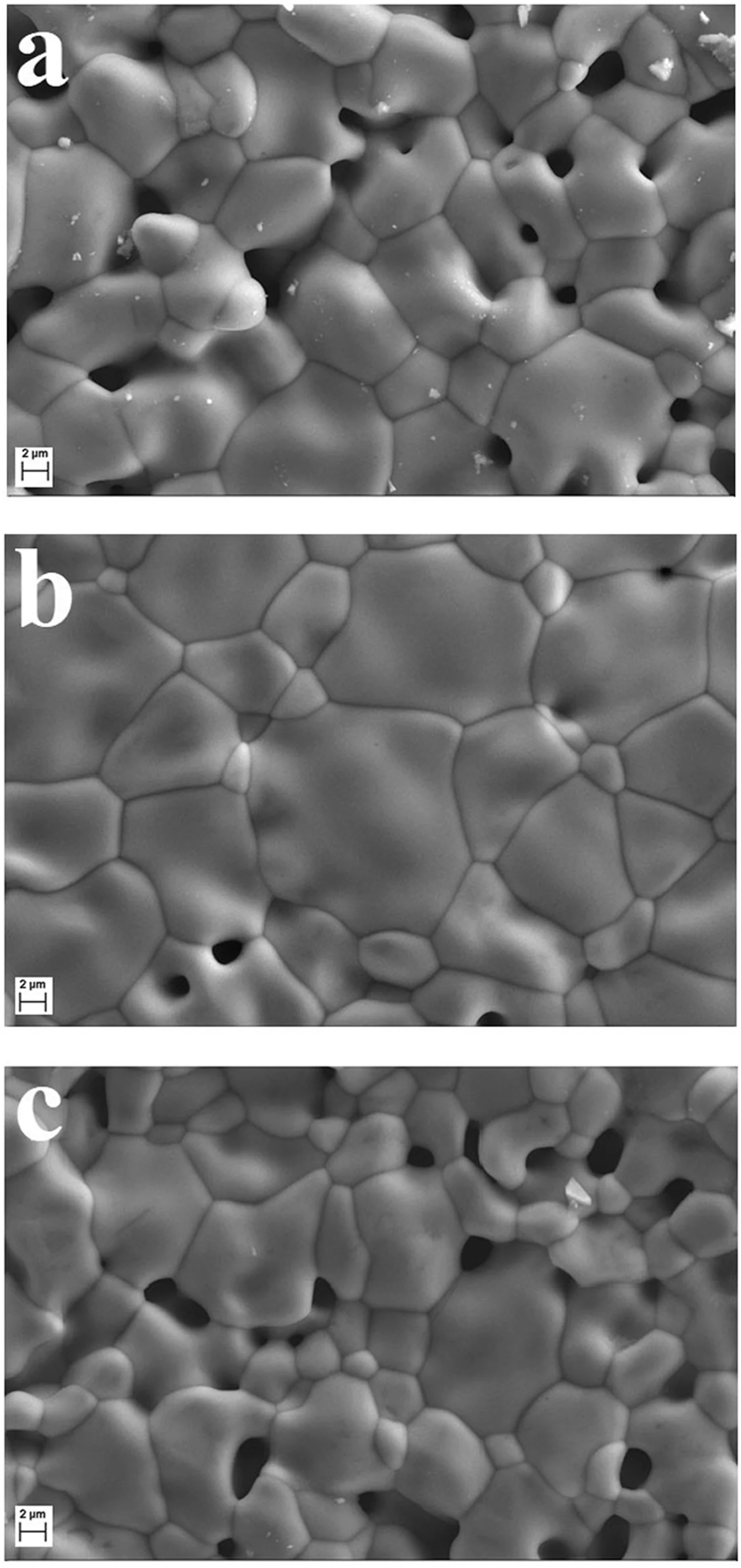
FESEM micrographs for (a) *x* = 0.00, (b) *x* = 0.04, and (c) *x* = 0.10 samples.

Figure 2 shows the temperature dependence of the electrical resistivity of the pristine CaMnO_3_ ceramics. As the temperature increases, the electrical resistivity continuously decreases, exhibiting a typical semiconducting behavior. The resistivity values remain relatively high over the entire investigated temperature range, reflecting the intrinsically low electrical conductivity of the undoped CaMnO_3_. For comparison, the inset presents the temperature-dependent resistivity of the representative Ce-doped composition (*x* = 0.04), which is replotted from our previous study.^19^ A remarkable reduction in electrical resistivity is observed upon Ce substitution, with the resistivity decreasing by several orders of magnitude throughout the measured temperature range. Furthermore, the electrical transport behavior changes from semiconducting in pristine CaMnO_3_ to metallic-like in the Ce-doped sample, where the resistivity gradually increases with an increase in temperature. These results clearly demonstrate the significant influence of Ce substitution on the electrical transport properties of the CaMnO_3_ systems. The pronounced decrease in the electrical resistivity can be attributed to the aliovalent substitution of Ce^4+^ for Ca^2+^, which introduces additional electrons into the CaMnO_3_ lattice. To preserve charge neutrality, a fraction of Mn^4+^ ions is reduced to Mn^3+^, resulting in the formation of mixed-valence Mn^3+^/Mn^4+^ pairs. These mixed-valence states facilitate electron hopping through the double-exchange mechanism, thereby markedly enhancing the electrical conductivity.^39,40^ Although the FESEM observations indicate noticeable changes in grain morphology and microstructure upon Ce substitution, these microstructural modifications are not sufficient to account for the several orders of magnitude decrease in the electrical resistivity. Instead, the dominant contribution arises from the electronic structure modifications associated with the increased Mn^3+^ concentration and the consequent enhancement of the Mn^3+^–O-Mn^4+^ double-exchange interaction, which governs charge transport in the Ce-doped CaMnO_3_ system. This transition from thermally activated semiconducting transport to a highly coupled metallic conduction pathway highlights the highly nonlinear characteristics of the data set, thereby presenting a robust benchmark for evaluating the forecasting capabilities of advanced regression models.

**Figure 2:**
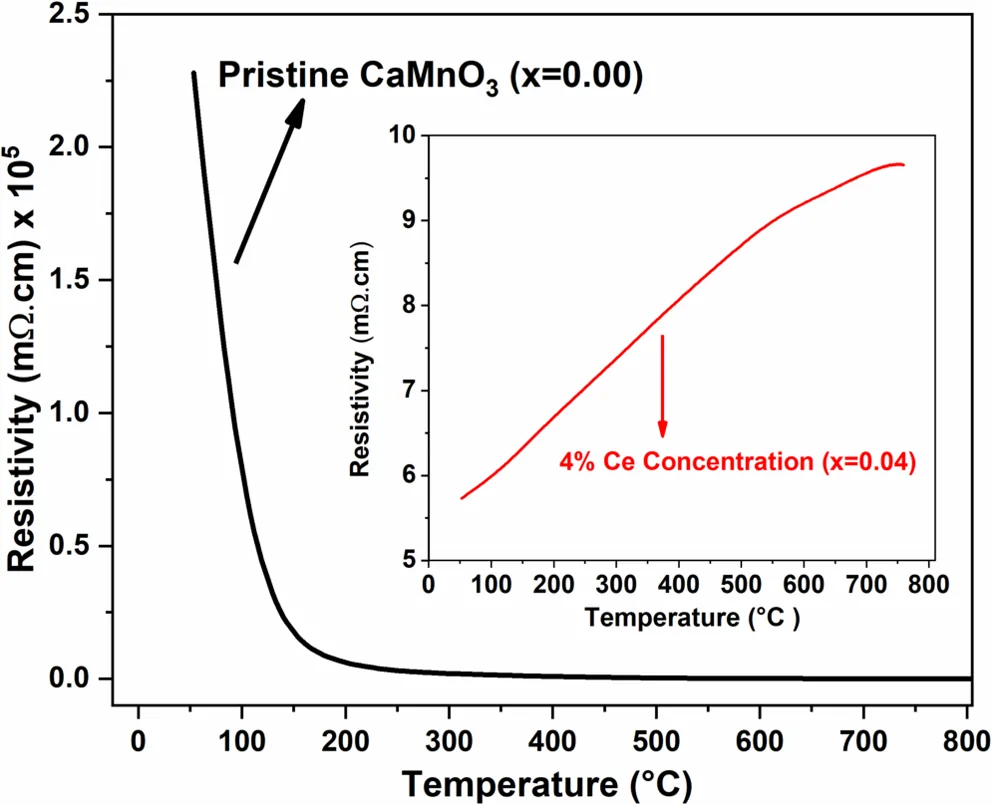
Resistivity measurements for *x* = 0.00 and 0.04.

Figure 3 shows the temperature dependence of the Seebeck coefficient of pristine CaMnO_3_. Throughout the entire investigated temperature range, the Seebeck coefficient remains negative, indicating that electrons are the dominant charge carriers and confirming the characteristic n-type semiconducting behavior of undoped CaMnO_3_. The pristine CaMnO_3_ sample exhibits relatively large negative Seebeck coefficient values, ranging from approximately −390 μV/K at low temperatures to about −245 μV/K at 800 °C. A broad minimum is observed around 430–450 °C, followed by a gradual decrease in the absolute Seebeck coefficient values with rising temperature. For comparison, the inset presents the temperature-dependent Seebeck coefficient of the representative Ce-doped composition (*x* = 0.06), which is replotted from our previous study.^19^ Notably, Ce substitution significantly reduces the absolute Seebeck coefficient over the entire temperature range. This reduction is attributed to the increased charge carrier (electron) concentration introduced by Ce doping. When Ce^4+^ ions substitute into Ca^2+^ sites, charge compensation occurs through the partial reduction of Mn^4+^ to Mn^3+^, thereby generating additional electrons. Consequently, the increased carrier concentration leads to a lower absolute Seebeck coefficient, which is consistent with the inverse relationship between carrier concentration and the Seebeck coefficient.^3^ The representative *x* = 0.06 composition is included solely for comparison to demonstrate the influence of Ce incorporation on the thermoelectric response. The complete Seebeck coefficient data set for all compositions was subsequently used to develop and validate the machine-learning models presented in this work. The explicit trade-off observed between the drastically reduced resistivity and the simultaneously diminished Seebeck coefficient underlines the classic thermoelectric optimization dilemma, highlighting the critical need for predictive data-driven frameworks to precisely identify the optimal doping thresholds.

**Figure 3:**
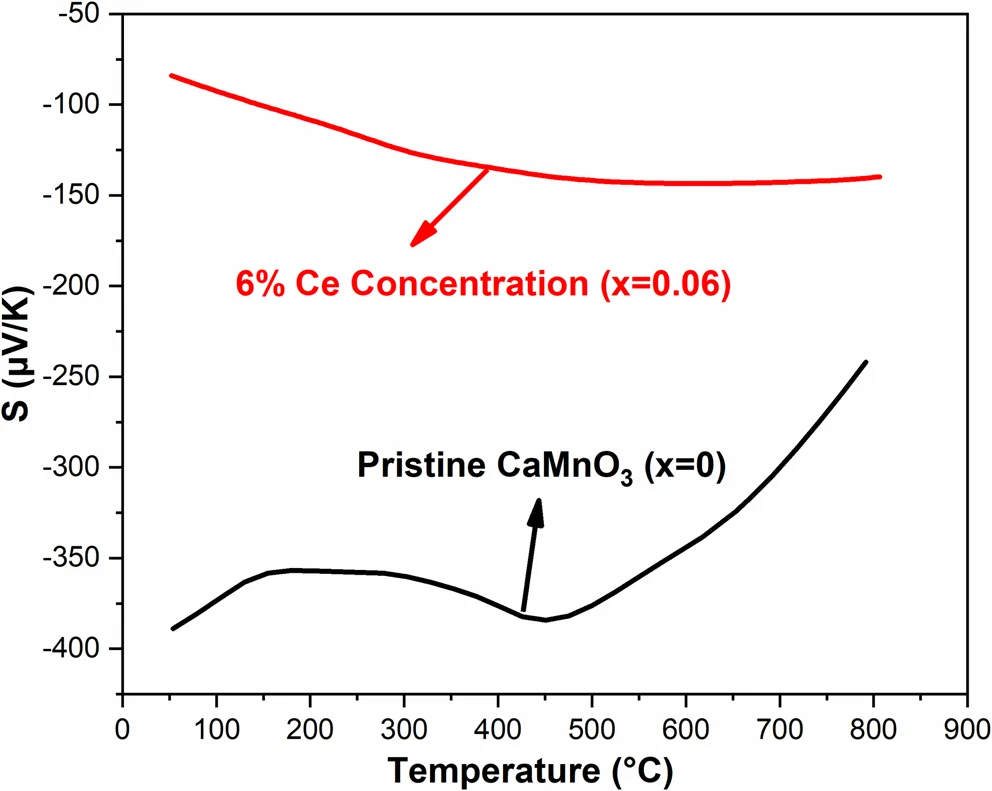
Seebeck coefficient for *x* = 0 and *x* = 0.06 samples.

### 3.2 Model Development and Optimization

The internal architectural configurations of the surrogate models were rigorously tuned via Bayesian optimization to establish a robust and reproducible predictive framework for the simultaneous mapping of electrical resistivity and the Seebeck coefficient. Relying on heuristic trial-and-error paradigms often yields suboptimal local minima and increases computational overhead. Conversely, the Bayesian approach models the objective function as a Gaussian process, sequentially exploiting and exploring the hyperparameter space to effectively minimize cross-validation loss. By evaluating the acquisition function over successive iterations, this framework dynamically balances the refinement of known optimal zones with the discovery of uncharted parameter regions, thus accelerating convergence toward global minima. The optimization search ranges, corresponding parameter spaces, and the definitively converged optimal values for the proposed hybrid CNN–LSTM framework alongside benchmark models such as SVR, MLP, and XGBoost are comprehensively summarized in Table 1.

**Table 1:** Search Ranges and Optimal Hyperparameter Configurations Obtained via Bayesian Optimization for the Evaluated Machine Learning Models

model architecture	hyperparameter	search range	optimized value
CNN–LSTM	initialLearnRate	[10^–4^, 10^–3^]	4.13 × 10^–4^
CNN–LSTM	numLSTMUnits	[10, 100]	11
CNN–LSTM	numConvFilters	[8, 64]	40
CNN–LSTM	dropoutRate	[0.1, 0.4]	0.100
CNN–LSTM	miniBatchSize	[4, 32]	27
SVM	BoxConstraint	[0.001, 1000]	41.56
SVM	KernelScale	[0.001, 1000]	13.29
MLP	LayerSizes	[1, 300]	5,8
MLP	InitialLearnRate	[10^–5^, 1]	Auto
XGBoost	NumLearningCycles	[10, 500]	10
XGBoost	LearnRate	[0.001, 1]	0.52

The comprehensive cross-validation results evaluating the predictive capabilities of the proposed hybrid CNN–LSTM framework against the optimized baseline architectures (SVR, MLP, and XGBoost) are presented in Table 2. The evaluation metrics reveal distinct differences in model performance, primarily driven by the ability of each architecture to handle the complex coupled transport behavior inherent in the thermoelectric properties.

**Table 2:** Comparative Predictive Performance of the Evaluated Machine Learning Models for Electrical Resistivity (RMSE and MAE in mΩ·cm) and Seebeck Coefficient (RMSE and MAE in μV/K) Prediction based on Cross-Validation Metrics

target property	model architecture	*R* ^2^ score	RMSE	MAE
Resistivity	CNN–LSTM (Proposed)	0.9982	0.1939	0.1410
Resistivity	SVM (Optimized)	0.5128	3.2246	2.2452
Resistivity	MLP (Optimized)	0.9303	1.2193	0.6944
Resistivity	XGBoost (Optimized)	0.9302	1.2205	0.6963
Seebeck Effect	CNN–LSTM (Proposed)	0.9987	1.8131	1.4549
Seebeck Effect	SVM (Optimized)	0.9404	12.2500	10.0149
Seebeck Effect	MLP (Optimized)	0.9389	12.4070	7.9148
Seebeck Effect	XGBoost (Optimized)	0.9412	12.1655	8.0271

For electrical resistivity, the proposed hybrid CNN–LSTM network achieves an exceptional *R* ^2^ score of 0.9982, accompanied by a remarkably low root-mean-square error (RMSE) of 0.1939 and a mean absolute error (MAE) of 0.1410. This stands in stark contrast to the support vector machine (SVM) model, which exhibits significant performance degradation, yielding an inadequate *R* ^2^ of 0.5128 and an elevated RMSE of 3.2246. The strict global optimization constraints and hyperplane dependency of SVR fail to capture the local fluctuations induced by Ce content variations on charge carrier mobility and density. While the Multilayer Perceptron (MLP) and XGBoost ensembles demonstrate competitive performance with *R* ^2^ values of 0.9303 and 0.9302, respectively, they generate nearly triple the error margin of the CNN–LSTM. This indicates that while standard shallow mapping and gradient tree boosting can track broad resistivity trends, they lack the spatial convolution required to reconstruct the exact boundaries of the composition–temperature landscape. The tracking of the Seebeck coefficient exposes further algorithmic nuances. All baseline models exhibit a high statistical correlation with *R* ^2^ values clustering tightly between 0.9389 and 0.9412. However, despite these strong correlation coefficients, SVM, MLP, and XGBoost suffer from high error magnitudes, with RMSE values scaling above 12.16. This anomaly points to a systematic variance where the baseline models capture the general direction of the thermal voltage gradient but miss individual structural transitions. The proposed CNN–LSTM resolves this limitation completely. It delivers an *R* ^2^ score of 0.9987 while simultaneously reducing the RMSE to 1.8131 and the MAE to 1.4549. The coupled integration of 1D convolutional layers to extract compositional features, joined with the recurrent tracking of the LSTM block to map continuous thermal gradients, enables the framework to predict sudden state changes in the Seebeck coefficient without introducing significant residual errors. The overall performance matrix confirms that the joint multioutput design of the CNN–LSTM framework effectively manages the physical trade-off between ρ and *S*. By learning the shared latent correlations within a single spatial-temporal sequence, the hybrid model prevents conflicting property predictions, yielding the highest reliability for subsequent power factor simulations. To rigorously evaluate the practical significance of this performance, the error magnitudes are contextualized across the entire operational range. For electrical resistivity, the RMSE of 0.1939 mΩ·cm corresponds to a relative variation of approximately 1.0% at the upper limit (19 mΩ·cm) and up to 5.5% at the lower boundary (3.5 mΩ·cm). Similarly, the Seebeck coefficient RMSE of 1.8131 μV/K yields a relative error of about 0.8% at high absolute magnitudes (|*S*| = 230 μV/K) and 3.6% at the lower bound (|*S*| = 50 μV/K). Because the power factor is computed from two independently predicted parameters as PF = *S* ^2^/ρ, the relative errors propagate according to the relation $\frac{\delta \mathrm{PF}}{\mathrm{PF}}\approx \frac{2\delta S}{S}+\frac{\delta \rho }{\rho }$ . Evaluating this error propagation within the identified optimum operational region (ρ ≈ 4.5 mΩ·cm, |*S*| ≈ 140 μV/K) results in a cumulative model uncertainty of approximately 7% (corresponding to ±0.0245 mW/K^2^m) on the reported peak power factor value of 0.35 mW/K^2^m. Although this uncertainty interval spans neighboring compositional responses, the model predictions indicate a favorable performance region around *x* = 0.05–0.06 relative to the extreme compositional end points. Subsequently, the Monte Carlo analysis involving 150,000 statistical iterations was used to further examine the response surface and identify the favorable composition–temperature region.

### 3.3 Prediction Performance Comparison

The multipanel scatter matrix presented in Figure 4 illustrates the predictive alignment for electrical resistivity across the evaluated model architectures, highlighting a distinct experimental data gap between 10 mΩ·cm and 14 mΩ·cm that naturally arises from the sharp drop in electrical resistivity as the Ce concentration increases between Ce_0.02_Ca_0.98_MnO_3_ and Ce_0.04_Ca_0.96_MnO_3_ compositions. Within this challenging topology, the proposed hybrid CNN–LSTM framework maintains an exceptional tracking capacity, binding the data points almost perfectly along the 1:1 identity line without showing sensitivity to the missing intermediate regime. This structural stability confirms that the joint spatial convolutions and sequential layer tracking prevent the model from generating erratic interpolations or numerical divergence within unmapped physical domains. By capturing the underlying continuous physical correlations rather than merely performing local numerical fitting, the deep hybrid architecture safely bridges these experimental data discontinuities.

**Figure 4:**
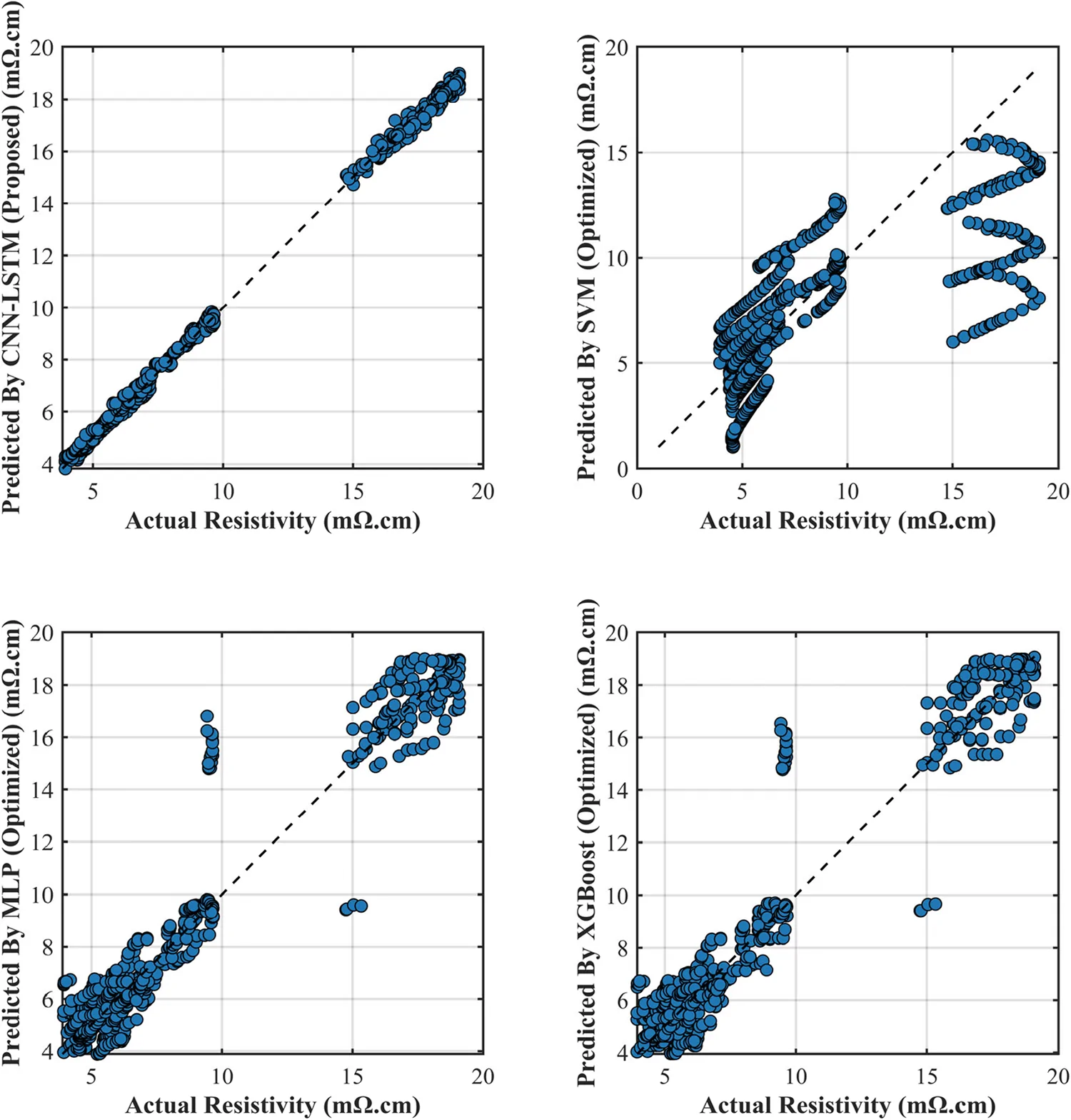
Multipanel scatter plots mapping the experimental versus predicted electrical resistivity (ρ) values across the evaluated model architectures (hybrid CNN–LSTM, SVM, MLP, and XGBoost). The dashed diagonal line represents the ideal 1:1 identity line.

Conversely, the baseline architectures suffer from severe tracking distortions when confronting both the data gap and local nonlinearities. The SVM profile in Figure 4 exhibits catastrophic branching, settling into systematic sinusoidal waveforms that severely overshoot or undershoot the diagonal axis, particularly in the high-resistivity zone above 15 mohm·cm. This massive distortion reflects the inability of the global kernel optimization to anchor a reliable regression hyperplane when a wide phase or compositional gap is present in the data set. Meanwhile, the MLP and XGBoost models demonstrate nearly identical behaviors, characterized by dense clustering in the lower region and structured dispersion in the upper regime. Both architectures exhibit notable out-of-boundary artifacts, predicting isolated vertical data structures near 9.5 mΩ·cm and 15 mΩ·cm that drift vertically away from the identity line. This clear dispersion pattern proves that standard feed-forward networks and gradient boosting trees tend to struggle with local transport property transitions when operating without the specialized spatial filtering mechanism embedded in the CNN–LSTM network.

The geometric distribution of predicted versus experimental observations further clarifies the computational behavior of each architecture, as evidenced by the multipanel scatter matrix presented in Figure 5 for the Seebeck coefficient. The proposed hybrid CNN–LSTM framework demonstrates near-ideal convergence, with data points tightly tracking the dashed 1:1 identity line across the entire continuum from −250 μV/K to −50 μV/K. This minimal dispersion along the diagonal demonstrates the efficacy of the joint spatial-temporal feature extraction mechanism, ensuring that both lower and upper experimental extremes are reproduced with high fidelity. In stark contrast, the benchmark architectures exhibit distinct nonphysical artifacts and structural limitations during the mapping process. SVM profile displays a critical structural anomaly characterized by distinct, branch-like segmentations and localized discontinuities rather than tracking a smooth continuous transformation. This step-like parallel trajectory stems from the global optimization constraints of the RBF kernel under a strict box constraint, which lacks the parametric flexibility needed to resolve localized nonlinear transitions. Consequently, while the global statistical metric (*R* ^*2*^) reflects a strong macro-level correlation by tracking the overarching trend, these localized structural artifacts and parallel trajectories account for the simultaneously elevated error margins (RMSE and MAE). Meanwhile, the MLP and XGBoost models demonstrate nearly identical scatter topologies, with an expansive, noisy distribution around the diagonal axis. While both frameworks successfully anchor their global predictions along the correct path, preventing the systemic branching observed in SVR, they introduce substantial variance in the intermediate transition zone between −150 μV/K and −50 μV/K. This noticeable dispersion indicates that standard feed-forward networks and iterative decision trees struggle to isolate subtle fluctuations governed by coupled transport physics without a specialized spatial filtering component.

**Figure 5:**
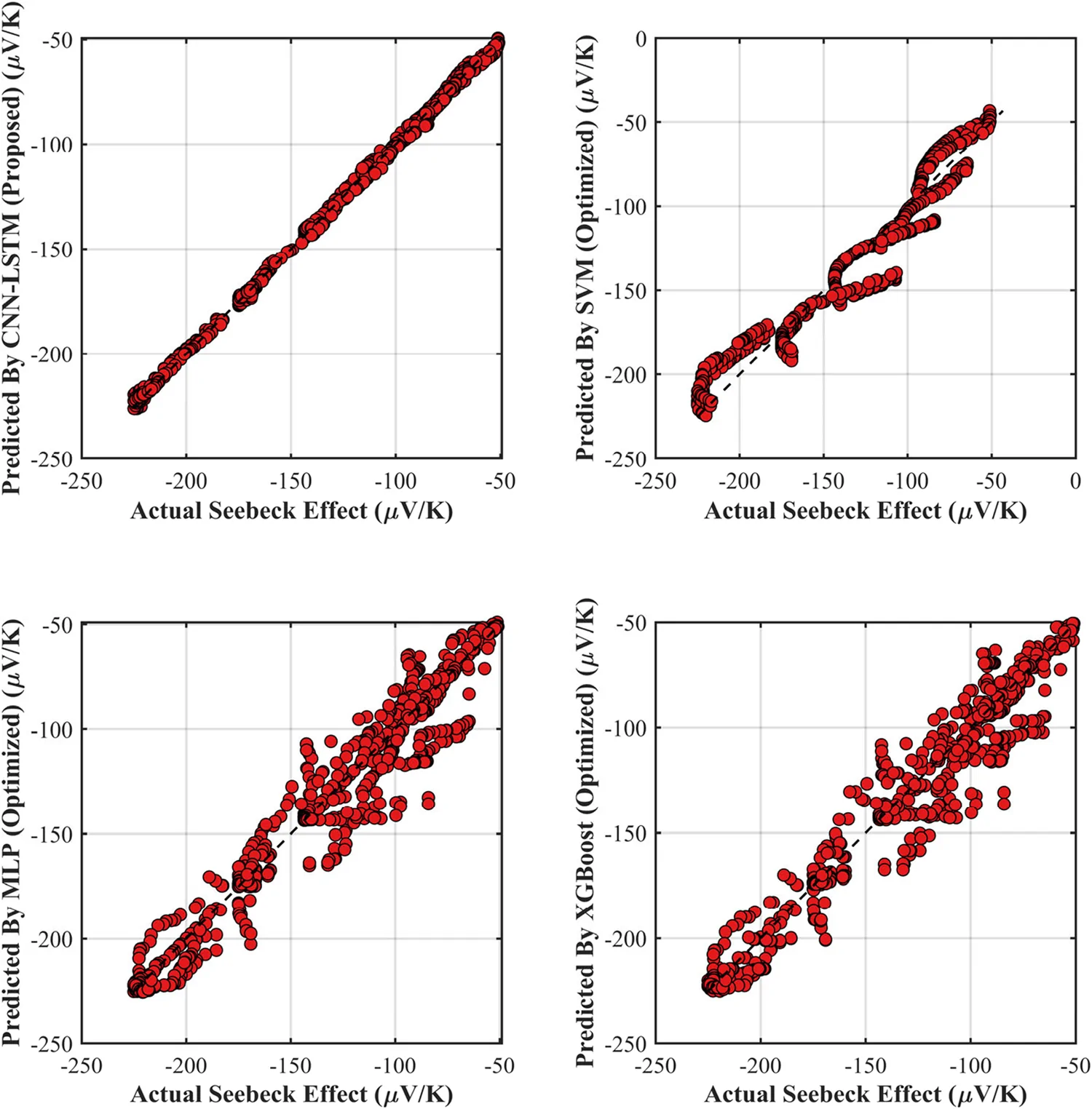
Multipanel scatter plots mapping the actual versus predicted values of the Seebeck coefficient across the evaluated model architectures (hybrid CNN–LSTM, SVM, MLP, and XGBoost). The dashed line represents the ideal 1:1 identity line.

The predictive performance of the machine-learning models was further evaluated using Taylor diagrams, which simultaneously compare the correlation coefficient (*R*), the root-mean-square (RMS) error, and the standard deviation between the experimental and predicted data sets.^41,42^ As shown in Figure 6a, the Taylor diagram for electrical resistivity indicates that the proposed CNN–LSTM model is located closest to the experimental reference point, demonstrating excellent agreement with the measured data. The model achieves a correlation coefficient (*R*) greater than 0.999 (which aligns with the coefficient of determination *R* ^2^ = 0.9982 listed in Table 2) while exhibiting a negligible centered RMS error and a standard deviation nearly identical to that of the experimental data set (≈4.65), indicating its ability to accurately reproduce both the overall trend and data variability. In contrast, the conventional machine-learning models show lower predictive performance. The MLP and XGBoost models exhibit similar statistical characteristics, with correlation coefficients of approximately 0.965 and standard deviations close to 4.55. Although these models provide reasonable predictions, their larger RMS errors indicate a reduced agreement with the experimental data compared with the CNN–LSTM model. The SVM model shows the weakest performance, with a correlation coefficient below 0.720 and a substantially lower standard deviation of approximately 3.15. This indicates that SVM fails to capture the full variability of the experimental resistivity data, resulting in oversmoothed predictions. A similar trend is observed for the Seebeck coefficient, as illustrated in Figure 6b. The experimental data set has a standard deviation of approximately 50.1, and the CNN–LSTM model again lies very close to the reference point, achieving a correlation coefficient (*R*) above 0.999 with a negligible centered RMS error. These results demonstrate that the proposed model accurately reproduces both the magnitude and variation of the experimental Seebeck coefficient. By comparison, the SVM, MLP, and XGBoost models form a closely grouped cluster with correlation coefficients between 0.968 and 0.970 and standard deviations of approximately 48.5. Although these models exhibit relatively high correlations with the experimental data, their greater distance from the reference point indicates larger prediction errors and a slight underestimation of the experimental variability. Overall, the Taylor diagrams clearly demonstrate that the hybrid CNN–LSTM model provides the most accurate and statistically consistent predictions for both electrical resistivity and the Seebeck coefficient.

**Figure 6:**
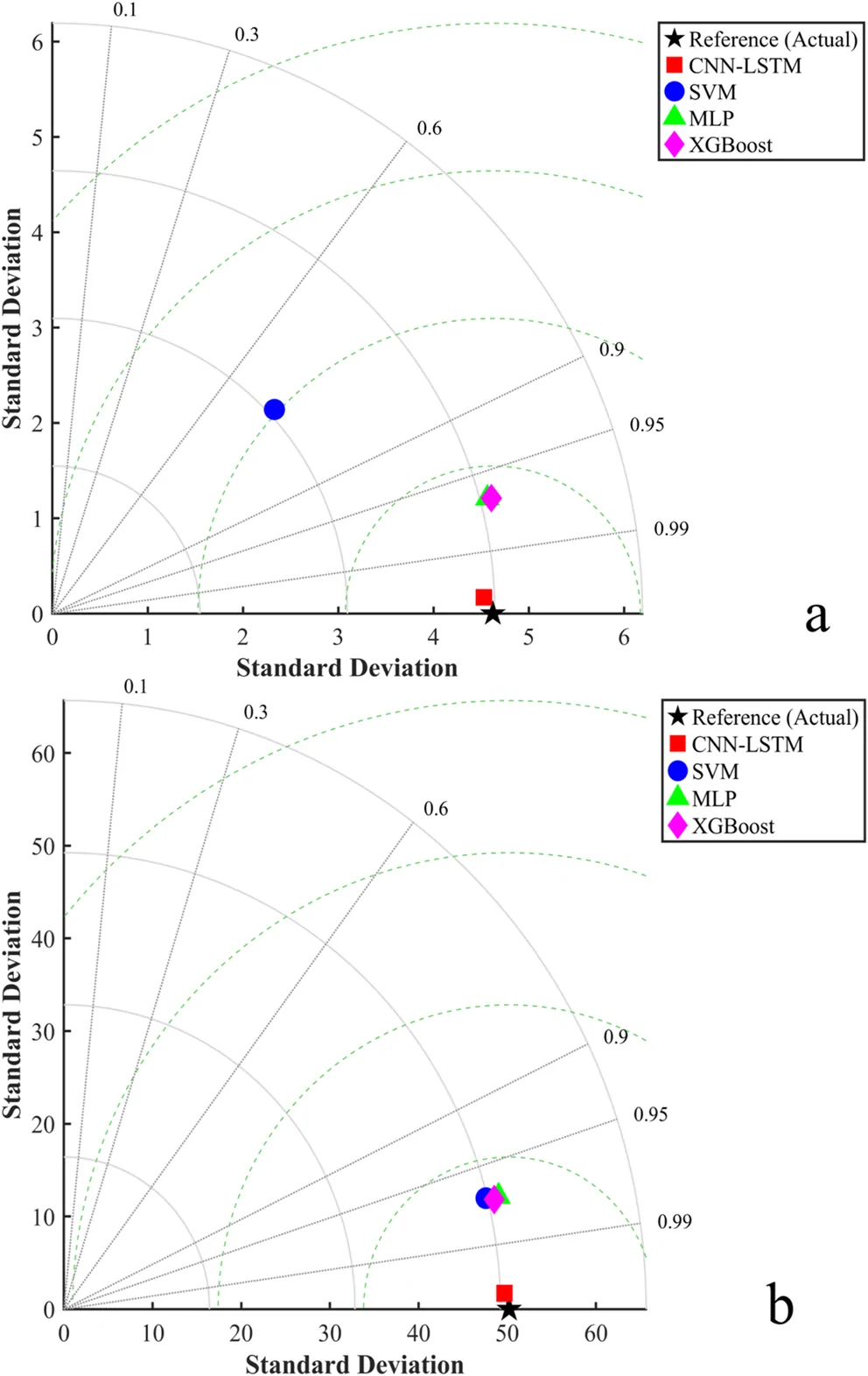
Taylor diagram illustrating the statistical correspondence between the experimental and predicted values of (a) electrical resistivity and (b) Seebeck coefficient.

### 3.4 Feature Importance and Monte Carlo Simulation for Power Factor

The relative importance of the input variables was evaluated using the permutation-based beeswarm plot shown in Figure 7, which illustrates both the global importance of each feature and the distribution of its contribution across all predictions. As shown in Figure 7, Ce content (%) exhibits substantially higher importance than temperature (°C), indicating that compositional variation is the dominant factor influencing the prediction of the thermoelectric transport properties. The impact values associated with Ce content are distributed over a considerably wider range, with several samples reaching absolute importance values close to 2.0, whereas the majority of temperature impacts remain below approximately 0.7. Moreover, the highest-impact Ce samples are predominantly associated with lower normalized feature values, suggesting that small compositional variations in the low-doping region have a pronounced influence on the predicted transport response. In contrast, temperature exhibits a narrower and more uniform importance distribution, indicating a comparatively stable contribution throughout the investigated temperature range. Overall, the permutation-based beeswarm analysis indicates that Ce content is the dominant predictor governing the model output, whereas temperature provides a secondary but complementary contribution to the prediction of the thermoelectric transport properties. These findings are in good agreement with the experimental observations, supporting the dominant role of Ce substitution in governing carrier concentration and the resulting electrical transport behavior in Ce-doped CaMnO_3_.^37,43^

**Figure 7:**
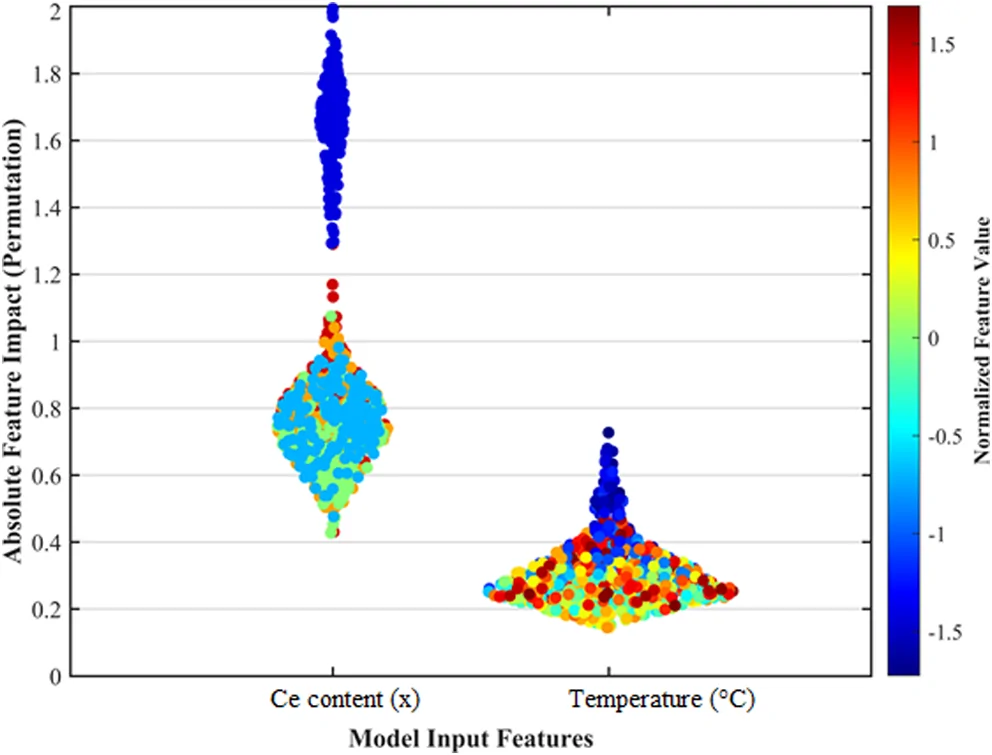
Permutation-based beeswarm plot showing the contribution of Ce content and temperature to the prediction of the thermoelectric transport properties using the optimized CNN–LSTM model.

To examine the composition–temperature dependence of the thermoelectric properties, 150,000 Monte Carlo simulations were performed using the trained CNN–LSTM model across a broad operational domain, with Ce content ranging from 0.02 to 0.10 and temperatures spanning from 50 to 800 °C. The resulting continuous multidimensional response surfaces and contour plots were generated using interpolation with cubic splines to ensure smooth and accurate data representation. The electrical resistivity response surface (Figure 8) reveals a pronounced nonlinear dependence on Ce concentration. At low Ce contents (≈0.02), the resistivity remains relatively high throughout the investigated temperature range, reaching values above 17 mΩ·cm, which reflects the intrinsically poor electrical conductivity of pristine CaMnO_3_. With increasing Ce concentration, resistivity decreases rapidly and a broad low-resistivity region develops between approximately 0.05 and 0.07 Ce content, where resistivity remains below 5 mΩ·cm over a wide temperature interval. This behavior originates from the donor nature of Ce^4+^ ions substituting Ca^2+^ sites. To preserve charge neutrality, part of the Mn^4+^ ions are reduced to Mn^3+^, thereby increasing the electron concentration and enhancing small-polaron hopping between Mn^3+^ and Mn^4+^ sites, which significantly improves electrical conductivity.^43,44^ At higher Ce concentrations, the resistivity reduction becomes less significant, suggesting that the carrier concentration approaches saturation and additional Ce incorporation provides only marginal improvement in charge transport. Similar transport characteristics have been reported for rare-earth-substituted CaMnO_3_ thermoelectric oxides, where moderate donor doping effectively suppresses electrical resistivity while preserving structural stability.^44,45^ The simulated Seebeck coefficient contour (Figure 9) exhibits an opposite tendency, demonstrating the well-known trade-off between electrical conductivity and thermopower. Low Ce concentrations produce relatively large negative Seebeck coefficients, approaching −220 μV/K, whereas increasing Ce incorporation gradually reduces the absolute Seebeck magnitude to approximately −60 μV/K at the highest simulated compositions. This behavior is expected because donor doping increases the electron carrier concentration, shifting the Fermi level closer to the conduction band and consequently reducing the entropy transported per carrier.^3^ Unlike the resistivity contour, the Seebeck surface displays a stronger simultaneous dependence on both temperature and composition, indicating that thermal excitation and carrier concentration jointly govern the evolution of thermopower. Such behavior follows the classical Pisarenko relationship, which predicts an inverse correlation between carrier concentration and the Seebeck coefficient in thermoelectric semiconductors.^3,44^ The combined influence of these competing transport parameters is reflected in the power factor response surface presented in Figure 10. Since the power factor depends on both the Seebeck coefficient and the electrical resistivity, neither the lowest resistivity nor the largest Seebeck coefficient independently produces the highest thermoelectric performance. Instead, the contour map indicates a favorable thermoelectric region around 0.05–0.06 Ce content and temperatures between approximately 350 and 500 °C, where the simulated power factor exceeds 0.35 mW/K^2^m. In this compositional window, the electrical resistivity has already been substantially reduced while the Seebeck coefficient still maintains a sufficiently large magnitude, leading to an optimal balance between the two competing transport parameters. At lower Ce concentrations, the high resistivity dominates the transport behavior despite the relatively large Seebeck coefficient, whereas excessive Ce incorporation further decreases the Seebeck coefficient and offsets the benefit of improved electrical conductivity, resulting in a reduction of the power factor. The relatively narrow high-performance region indicates that the thermoelectric response is sensitive to Ce concentration, highlighting the importance of carefully controlling the doping level. The hybrid CNN–LSTM model combined with Monte Carlo simulations successfully captures these complex nonlinear interactions and provides a continuous design map for examining favorable composition–temperature conditions, thereby providing a data-driven means of screening the investigated composition–temperature space without relying solely on conventional trial-and-error experiments. Finally, it should be acknowledged that while *x* = 0.06 is part of our experimentally synthesized data set, the favorable region extending toward *x* = 0.05 and the corresponding temperature range of 350–500 °C are model-based predictions and should be considered within the uncertainty associated with the model predictions and experimental measurements. Experimental validation of this predicted region would therefore be required to establish its practical thermoelectric performance.

**Figure 8:**
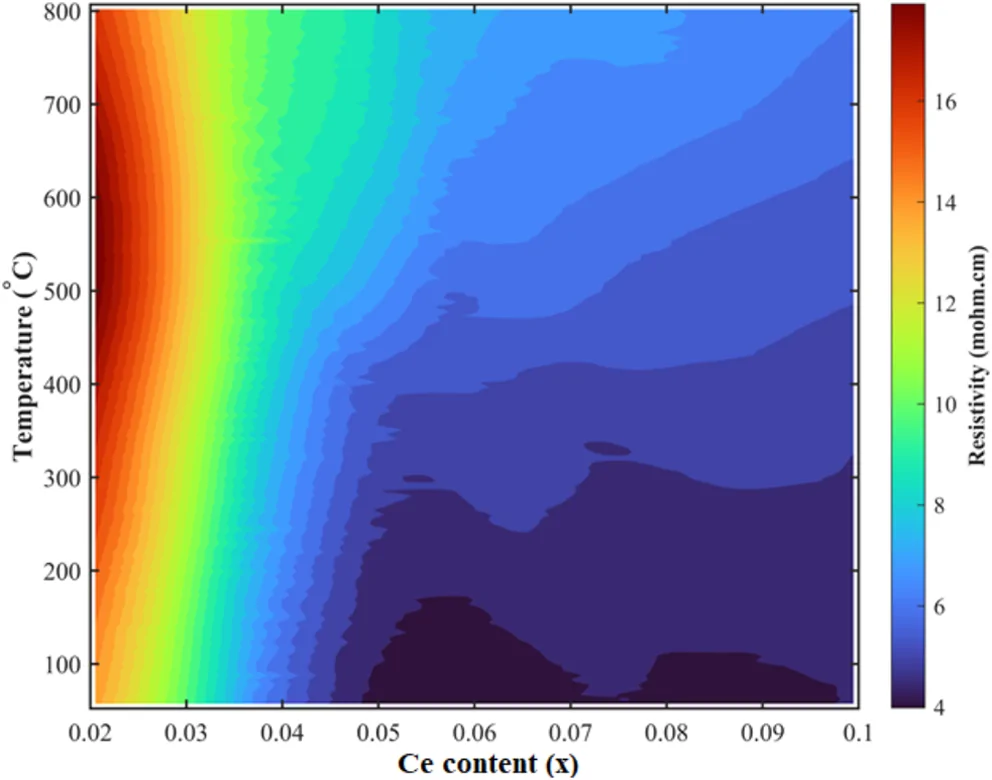
Monte Carlo simulation-based contour map of the electrical resistivity as a function of Ce concentration and temperature, predicted using the hybrid CNN–LSTM model.

**Figure 9:**
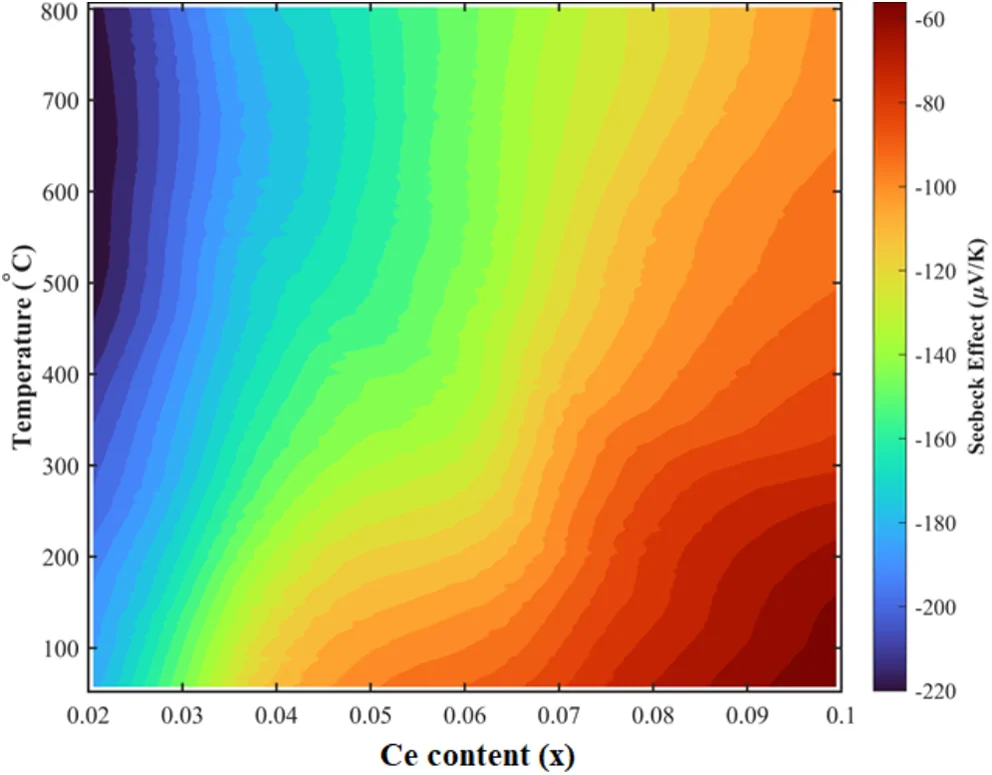
Monte Carlo simulation-based contour map of the Seebeck coefficient as a function of Ce concentration and temperature, predicted using the hybrid CNN–LSTM model.

**Figure 10:**
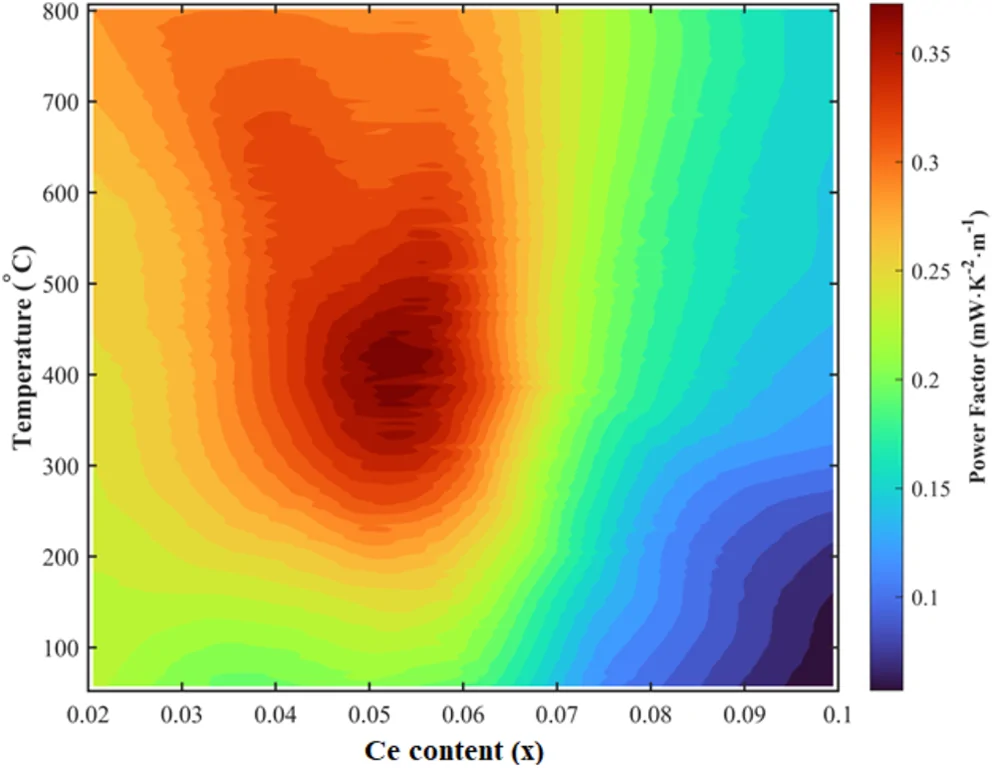
Monte Carlo simulation-based contour map of the thermoelectric power factor as a function of Ce concentration and temperature, predicted using the hybrid CNN–LSTM model.

To further evaluate the generalization capability of the hybrid CNN–LSTM framework across composition, a Leave-One-Composition-Out (LOCO) validation was conducted. In this approach, the entire data set corresponding to the nominal Ce concentration *x* = 0.06 was completely withheld from the training phase and used exclusively for independent testing. The model was trained using the remaining compositions and then used to predict the transport properties of the withheld composition. As shown in Figure 11, the LOCO validation yielded R^2^ = 0.9153 for electrical resistivity and *R* ^2^ = 0.9869 for the Seebeck coefficient. The corresponding RMSE and MAE values were 0.2957 mΩ·cm and 0.2417 mΩ·cm for electrical resistivity, and 2.0904 μV/K and 1.6810 μV/K for the Seebeck coefficient, respectively. These results demonstrate that the hybrid CNN–LSTM model maintains good predictive capability for a composition not included during training and provide additional support for its application across intermediate composition ranges.

**Figure 11:**
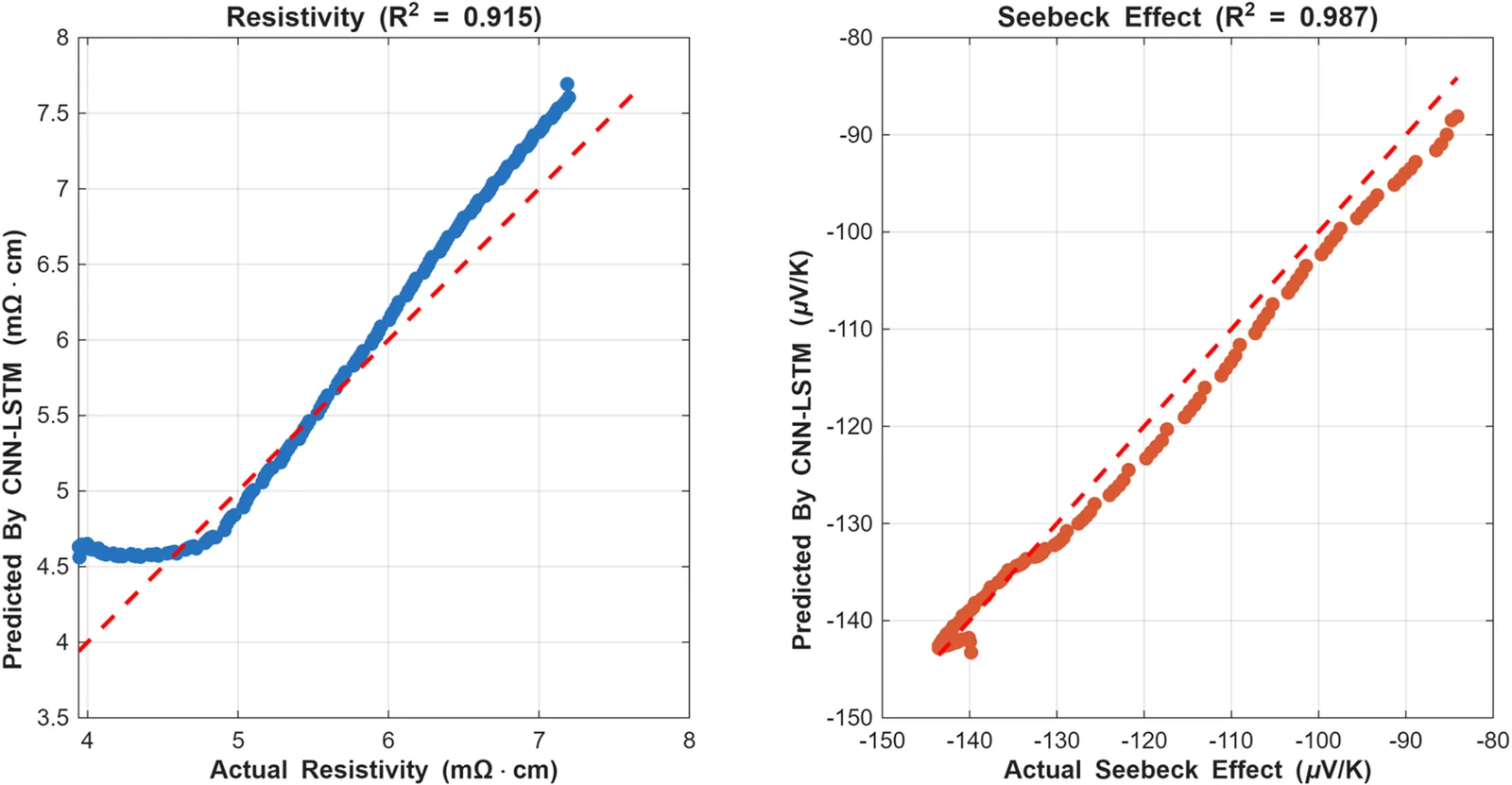
LOCO validation results for the hybrid CNN–LSTM model at the withheld Ce composition of *x* = 0.06: experimental versus predicted values for electrical resistivity and Seebeck coefficient.

## 4 Conclusion

In this study, a comprehensive data-driven framework combining machine learning techniques with Monte Carlo simulations was developed to predict and optimize the thermoelectric performance of Ce-doped CaMnO_3_ ceramics. Experimental electrical resistivity and Seebeck coefficient data measured over the temperature range of 50–800 °C were employed to train and evaluate four predictive models: SVM, MLP, XGBoost, and a hybrid CNN–LSTM network. Among the investigated models, the hybrid CNN–LSTM architecture achieved the highest predictive accuracy, yielding *R* ^2^ values of 0.9982 for electrical resistivity and 0.9987 for the Seebeck coefficient. The model successfully captured the nonlinear relationships between composition, temperature, and transport properties, providing more reliable predictions than the conventional machine learning algorithms. In addition, LOCO validation, in which the *x* = 0.06 composition was excluded from training and used for independent testing, provided further evidence of the model’s capability to generalize to a composition not included during training.

Permutation feature importance analysis indicated that the Ce concentration is the dominant parameter governing the evolution of the transport properties, whereas temperature primarily modulates their magnitude within the specific composition and temperature ranges investigated in this study. This finding highlights the critical role of compositional engineering in improving the thermoelectric performance of the Ce-doped CaMnO_3_ ceramics.

The trained CNN–LSTM model was subsequently combined with 150,000 Monte Carlo simulations to construct multidimensional response surfaces and identify the optimum thermoelectric operating region. The optimization results revealed that a Ce content of *x* = 0.05–0.06 and temperatures between 350 and 500 °C provide the most favorable combination of electrical resistivity and Seebeck coefficient, resulting in a simulated power factor exceeding your specified value.

Overall, the proposed framework provides a data-driven approach for analyzing the composition–temperature dependence of thermoelectric transport properties and identifying favorable operating regions in Ce-doped CaMnO_3_ ceramics while complementing experimental investigations under the conditions explored in this study.

## Data Availability

The experimental thermoelectric data (electrical resistivity, Seebeck coefficient, and power factor) generated during this study are openly available in the Zenodo repository at https://zenodo.org/records/22723698?token=eyJhbGciOiJIUzUxMiJ9.eyJpZCI6IjE5YTRjMzk4LWIyNGMtNDNlYy04ZGZhLTFhODNjNDFiYWZkNyIsImRhdGEiOnt9LCJyYW5kb20iOiIyMDlkNmYxNTlmNWRmNzEyMGJlM2IyODkyNDE1ODFlZCJ9.aYGAJX0rr_bc3SN5Kqmoq8kSAs8bD13yINzfhEp-fAm2N0tsag9BtIntBqYC_4Te8U4SlohJFN5yzQsrL3r7rg
